# Brain Health across the Lifespan: A Systematic Review on the Role of Omega-3 Fatty Acid Supplements

**DOI:** 10.3390/nu10081094

**Published:** 2018-08-15

**Authors:** Emma Derbyshire

**Affiliations:** Nutritional Insight Limited, Surrey KT17 2AA, UK; emma@nutritional-insight.co.uk; Tel.: +44-(0)-758-437-5246

**Keywords:** brain health, omega-3 fatty acids, cognition, learning, mindfulness

## Abstract

The brain is the most significant and complex organ of the human body. Increasingly, we are becoming aware that certain nutrients may help to safeguard brain health. An expanse of research has investigated the effects of omega fatty acids in relation to brain health but effects across the lifespan have not been widely evaluated. The present systematic review collated evidence from 25 randomized controlled trials (*n* = 3633) published since 2013. Compared with control groups, omega-3 supplementation generally correlated with improvements in blood biomarkers. Subsequently, these appear to benefit those with lower baseline fatty acid levels, who are breastfeeding or who have neuropsychiatric conditions. Whilst multiple studies indicate that omega fatty acids can protect against neurodegeneration in older adults, more work is needed in the years preceding the diagnosis of such medical conditions. Bearing in mind the scale of ageing populations and rising healthcare costs linked to poor brain health, omega supplementation could be a useful strategy for helping to augment dietary intakes and support brain health across the lifespan. Ongoing research is now needed using harmonious methodologies, supplement dosages, ratios and intervention periods to help formulate congruent conclusions.

## 1. Introduction

The brain is one of the body’s most complex organs and indeed one of its most crucial organs. Throughout the lifespan, the brain helps to make sense of the world, oversee daily operations and life itself [1]. Brain health is a multi-dimensional concept but, overall, refers to the ability to concentrate, remember, learn, play and maintain a clear and active mind [1]. The US Centers for Disease Control and Prevention defines a healthy brain as: “One that can perform all the mental processes that are collectively known as cognition, including the ability to learn new things, intuition, judgment, language, and remembering” [2].

We now recognize that a healthy brain is needed to live a fuller and longer life. Brain health supports human thoughts, planned actions and emotional connections [3]. Together these can influence the daily lives and indeed “quality” of lives of individuals, families and communities [3]. Maintaining brain health across the course of a lifetime can also help to optimize levels of independence [3]. Preserving brain health is also important in the mission to curtail heavy healthcare and economic costs that poor brain health can bring [3]. Over time, poor brain health can manifest in several different ways but characteristically as cognitive impairment or dementia [4]. A host of underlying cellular and molecular changes within the brain including oxidative damage, mitochondrial impairment, and alterations in glucose-energy metabolism and nerve inflammation have been purported to contribute to cognitive decline and brain ageing [5].

In the UK alone the cost of dementia is estimated to more than double in the next 25 years from £26 billion to £55 billion by 2040 [6]. From a global perspective, there are more than 9.9 million new cases of dementia each year equating to one new case every 3.2 seconds [7]. Rates are also climbing in China, India, South Asia and the Western Pacific, an upward trend fuelled by ageing populations in these regions [7]. Meta-analytical evidence from clinical trials and observational studies showed that >1 g/day docosahexaenoic acid/eicosapentaenoic acid (DHA/EPA) significantly improved episodic memory in older adults with a history of mild memory complaints [8]. Other “gold-standard” meta-analytical work has found that blood levels of EPA, DHA and total omega-3 fatty acids were significantly reduced amongst individuals with dementia - EPA was reduced amongst those with “predementia” compared with healthy elderly controls [9]. These are important findings signifying the role of omea-3 fatty acids in the pathophysiology of dementia with EPA also appearing to potentially be a useful marker of cognitive impairment.

Lamentably, mental health problems are another major cause of the disease burden globally [10]. This condition alone is now estimated to contribute to more than 40 million years of disability in 20- to 29-year-olds [11]. Bearing this in mind, we should not foresee poor brain health as a geriatric condition. Signs of cognitive decline can be evident in adults as young as their twenties [12]. Rates of cognitive deterioration have been found to accelerate in the 15 years preceding death—this includes 3 to 7 years before mild cognitive impairment diagnosis and to 1–11 years before dementia is diagnosed [13]. The causes of poor brain health are multi-factorial, quite often having the same modifiable risk factors as cardiovascular disease such as obesity, physical inactivity, smoking, diabetes mellitus and depression [14]. Alongside this, a growing body of evidence suggests that certain nutrients may help to normalize or even attenuate some of the changes that occur with brain ageing [5,15]. For example, research amongst patients with Alzheimer’s disease shows that brain nutrient status parallels circulatory status, indicating that the brain dependents on certain nutrients from the circulation [16]. DHA is an example of one such nutrient with this being abundant in neuronal membranes [17]. Quantitatively, DHA is the most important omega-3 fatty acid in the brain though there is now a growing body of evidence that others including EPA and docosapentaenoic acid (DPA) also possess neuroprotective properties most likely having both independent and shared effects [18].

Alpha-linoleic acid (ALA, omega-3) and linoleic acid (LA, omega-6) are essential fatty acids that are not produced in adequate amounts by humans [19]. For these reasons they need to be consumed from exogenous sources. Once ingested they produce other fatty acids at the two sides of metabolic pathway [20]. ALA yields EPA and DHA—omega-3 fatty acids. LA yields the omega-6 fatty acids gamma-linoleic acid (GLA) and arachidonic acid (ARA) [20]. EPA and DHA produce anti-inflammatory eicosanoids whilst the ARA side of the pathway generates eicosanoids that can exacerbate inflammation [19]. For instance, whilst we need some n-6 fatty acids for health, Western diets provide an overabundance of these which can displace DHA from membrane phospholipids [21]. Subsequently, proportionally higher amounts of omega-3 fatty acids are needed to protect aspects of health, including brain health [19]. A summary of the extended roles of omega-3 fatty acids in preventing non-communicable diseases is also shown in Table 1 [22]. Given this, the present systematic review focuses on the role of omega-3 supplements in relation to brain health across the lifespan.

## 2. Methods

A comprehensive literature search was conducted to identify randomized controlled trials (RCTs) examining the relationship between omega-3 fatty acid supplements and aspects of brain health. The US Centers for Disease Control and Prevention definition of a healthy brain formed the basis of the search terms used [2]. A PubMed search was undertaken to identify relevant RCTs using the selection filter. Filters were applied to identify English-language human trials published in the last 5 years (between January 2013 and May 2018). Search terms applied were: “omega” fatty acids AND “brain health”, “mindfulness”, “cognitive function”, “learning”, “judging”, “remembering”, “memory/memorizing” and “language use”.

Studies were organized according to the seven ages of the brain: (1) Pregnancy, (2) Infancy, (3) School age, (4) Teenage, (5) Young adults, (6) Middle age, (7) Older age/Dotage (Table 2). Studies focusing on school age or teenage subjects were merged in Tables Tables1 Tables2 due to overlapping age ranges. RCTs were included if the following criteria were met: (1) the trial was randomized and involved human subjects; (2) the trial was a controlled intervention using omega-3/6 supplements; (3) the trial clearly specified the dose and form of fatty acids; (4) the trial length was reported; (5) the trial was not a pilot study; (6) the trial did not use multi-domain interventions. Included participants did not have any severe, long-term medical conditions. Studies including participants with attention deficit hyperactivity disorder (ADHD), autism, depression and/or mild cognitive impairment (MCI) were included. When full texts were not available, these were purchased where sites permitted.

### Data Extraction

**Figure 1:**
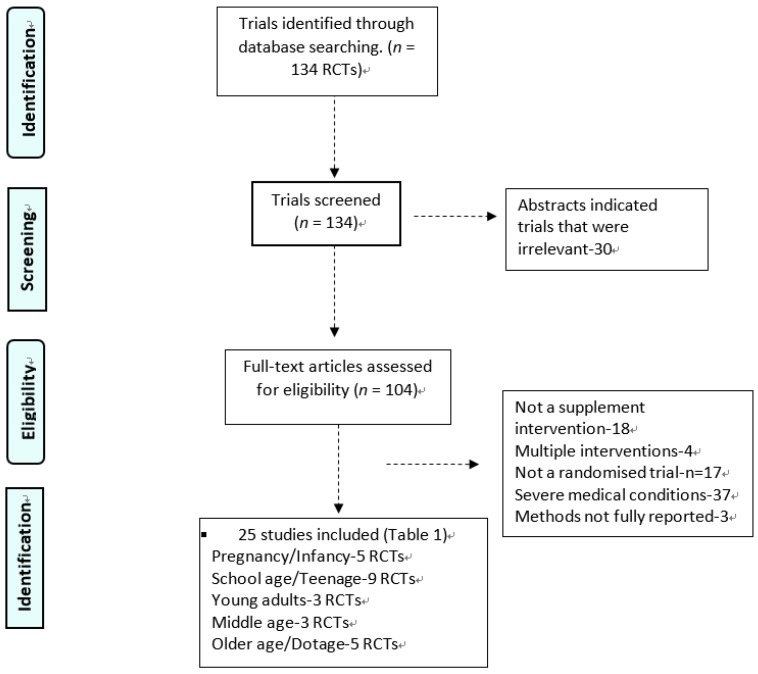
Algorithm for database search results.

The present systematic review followed the Preferred Reporting Items for Systematic Reviews and Meta-Analyses (PRISMA) statement [28]. Publications were excluded if PRISMA benchmarks were not included in the trial publication but instead covered elsewhere. As shown in Figure 1, abstracts and papers were evaluated for relevance. Once identified, all eligible RCT had relevant data extracted, including: author, year, location, life stage, sample size, age, gender, baseline health status, study design, form of intervention, aspect(s) of brain health studied and outcomes measured. Relevant data extracted from the studies included the dose and composition of fatty acid supplements, the randomization method, and withdrawal rates. The Jaded scale and criteria was then used to develop quality scores for each RCT [27]. Quality scores were graded between 1 and 5 with higher scores being indicative of higher quality (Table 3).

## 3. Results

The PubMed search identified 134 RCT papers after an adjustment for replica papers. Of these, 109 papers were discarded after reviewing the abstracts and article content as they did not meet the inclusion criteria. This left 25 RCT articles for general review. The algorithm of qualifying publications is shown in Figure 1. Of these, six studies were conducted in the United States or Canada, 11 in Europe or the United Kingdom, two in Asia and seven in Australasia.

### 3.1. Pregnancy and Infancy

Five RCTs considered the effects of omega supplementation during pregnancy or infancy in relation to neurodevelopment. The earliest trial began from 16 weeks of pregnancy until delivery allocating mothers to take 400 mg DHA/day [32]. When followed up at 14 to 18 months after delivery, scientists found that omega supplementation was associated with a reduced risk of poor language development compared with the placebo [32].

Other trials had less conclusive results. One study from 20 weeks of pregnancy using a higher 800 mg/day dose of DHA until birth did not observe any effects on infants’ attention or working memory [33]. Similarly, 400 mg EPA/DHA provided from the 28th week of pregnancy until the cessation of breastfeeding was not found to affect infant neurodevelopment although the DHA profile and levels of nervonic acid (nerve cell myelin fatty acids) improved [34].

In the postpartum and infancy period, one trial allocating 89 lactating women to 200 or 400 mg DHA daily over 6 weeks observed that maternal plasma and breast milk DHA levels increased which improved the ratio of fatty acids available to infants for infant brain development [36]. Other work forming part of the Childhood Asthma Prevention Study [30] found that consuming tuna fish oil daily (high in omega-3 fatty acids) for the first 5 years of life correlated with plasma omega-3 levels, literacy and numeracy performance at age 8 [30].

### 3.2. School Age and Teenage

A growing body of evidence has explored inter-relationships between omegas and brain health at this life stage. Nine RCTs met the inclusion criteria with four of these concentrating on children with Autistic Spectrum Disorder (ASD) or Attention Deficit Hyperactivity Disorder (ADHD) [29,31,35,49]. One trial included 7- to 14-year-olds with depression and/or bipolar [37] with the remainder recruiting healthy mainstream or children underperforming at school [38,39,40,41].

One trial replicating previous research on mainstream children (aged 7–9 years) underperforming in reading found that 600 mg/day of algal oil DHA over 16 weeks showed some minor differences in behavioral aspects but effects on learning were less apparent [38]. Authors concluded that findings may have been dissimilar to earlier research due to national curriculum changes, a larger recruitment area, and an underpowered sample size [38]. Other work on mainstream children at school has observed benefits. For example, a 3-month trial providing 9- to 10-year-olds with three Omega 3/6 capsules (Equazen®) twice daily (corresponding to a daily dose of 558 mg EPA, 174 mg DHA, and 60 mg gamma-linoleic acid) observed improvements in reading ability-particularly clinically relevant “phonologic decoding time” and “visual analysis time” compared with the placebo, with this being most evident in children with attention problems [39].

Five trials focused on children with mood or neuropsychiatric conditions. Amongst children aged 7 to 14 years with mood disorders, 2 g/day of omega fatty acids (1400 mg EPA and 200 mg DHA) over 12 weeks improved EPA levels by sevenfold and DHA levels by half [37]. EPA, in particular, correlated positively with mood responses and inversely with body weight indicating that smaller children could be more responsive to omega supplementation [37]. Amongst children and teenagers (*n* = 60) with ASD, omega-3 fatty acids (962 mg or 1155 mg daily) over 8 weeks improved the fatty acid composition of biological membranes and some markers of behavior although longer periods of follow up were needed to observe clinical changes [29]. A longer 16-week trial conducted on 40 boys (8–14 years) with and without ADHD found that 650 mg EPA/DHA provided via the ingestion of 10 g margarine daily enhanced parent-rated attention [31]. Improvements in spelling, attention, hyperactivity, cognitive problems and reduced oppositional behavior have also been observed in 6- to 13-year-olds with ADHD supplementing with omega fatty acids for up to 1 year [35]. Other work on 6- to 12-year-olds with ADHD showed that two daily capsules providing 720 mg omega-3 (600 mg EPA, 120 mg DHA) improved working memory function [49].

Amongst malnourished children, a 3-month Mexican trial found that three daily capsules of omega fatty acids (60 mg DHA and 90 mg EPA per capsule) taken by 8- to 12-year-olds improved 11 of 18 neuropsychological variables including processing speed, visual-motor coordination, perceptual integration, attention and executive function in more than 70% of the omega-3 supplemented children [40]. Similarly, amongst Australian children aged 3 to 13 years fish oil capsules (Equazen^®^) providing EPA (93 mg), DHA (29 mg) and GLA (10 mg) taken daily for up to 40 weeks improved measures of cognitive development after 20 weeks, particularly amongst 7- to 12-year-old indigenous children [43].

### 3.3. Young Adults

Three RCTs examined the role of omega fatty acids on brain health in young adults. A double-blind trial comprised of 72 healthy young adults (mean age 20 years) observed that 2800 mg/day fish oil (1680 mg EPA and 1120 mg DHA) over 35 days kept feelings of anger and confusion stable compared to increases in the placebo group [42]. A similar length 30-day trial where of 20- to 34-year-olds provided with varying ratios of EPA to DHA confirmed that EPA-rich supplementation enhanced neurocognitive function -implying that the brain worked less hard yet had better cognitive performance [46]. Other work using DHA specifically (1.16 g daily) over 6 months (mean age 33 years) observed that DHA significantly improved episodic memory in young women and reaction times of working memory in men, indicating benefits for those whose diets were habitually low in DHA [45].

### 3.4. Middle Age

Three RCTs were identified for this life stage. One 26-week trial providing 2200 mg/day omega-3 fatty acids to 50- to 75-year-olds (mean age 62 years) found that this dose significantly improved the recollection of object locations, implying positive effects on memory function [44]. Other research recruiting adults of a similar age range and time frame showed that 2200 mg/day omega-3 fatty acids improved brain structure, namely via white matter microstructural integrity and the volume of grey matter [48]. One trial focusing on adults with loneliness-related memory problems (mean age 51 years) discovered that omega-3 supplementation (1250 or 2500 mg/day) over 4 months attenuated verbal episodic memory declines that were linked to loneliness [47].

### 3.5. Older Age/Dotage 

Five RCTs have focused on brain health in later life. One RCT focusing on adults over the age of 70 years showed that 800 mg DHA and 225 mg EPA over 36 months helped to maintain executive function amongst those at risk of dementia with a low omega-3 index [50]. Amongst Chinese elderly people of a similar age (mean 71 years) with Mild Cognitive Impairment, 480 mg DHA and 720 mg EPA taken daily over 6-months significantly improved cognitive aptitude scores and working memory [51] compared with the olive oil placebo. A similar length trial conducted on older adults with subjective memory impairment showed that 2400 mg EPA + DHA significantly improved omega-3 red blood cell levels, working memory performance and brain signals during working memory challenges, indicating that neuronal response was heightened [52]. Some work with krill oil showed that omega-3 fatty acids may activate cognitive function in older adults which was determined by changes in cerebral cortex ox hemoglobin levels over 12 weeks [53]. Older adults provided with 800 mg DHA and a higher ratio of EPA-1200 mg had attenuated levels of oxidative stress -a risk factor thought to be implicated in the pathophysiology of depression and prevention of depressive symptoms [54].

## 4. Discussion

There is a strong body of evidence signifying that omega-3 fatty acids from dietary sources are under consumed. For example, systematic evidence from 53 studies across 17 different European countries shows that mean intakes of EPA and DHA are only consumed as recommended in 26% of countries, indicating that intakes are largely suboptimal [55]. Evidence from the US National Health and Nutrition Examination Survey (NHANES) survey similarly shows that childbearing aged women are eating seafood in amounts significantly lower than the Dietary Guidelines for Americans contributing to low habitual intakes of EPA and DHA [56].

Along with concerns over inadequate EPA and DHA intakes, interest in the “preservation” of brain health is emerging [3,55]. The American Heart Association/American Stroke Association has identified the risks of poor brain health as being similar and closely associated to those of cardiovascular disease [3]. Within their practical considerations, seven key ideal health behaviors are identified which include: not smoking, physical activity, a healthy body mass index (BMI), blood pressure, blood glucose, cholesterol levels and diet aligned with current healthy eating guidelines [3]. Increasingly, mindfulness is also being seen as a useful adjunctive therapy amongst those with ADHD [57,58] with this strategy also appearing to help reduce stress amongst older adults with memory complaints [59].

From a dietary perspective, findings from RCTs showed that omega fatty acid supplementation could help to reinforce habitual intakes by raising blood levels. For example, improvements in lipid profiles were observed in at least seven studies [30,34,36,37,49,52,53]. Certain subgroups such as those with lower baseline blood fatty acids levels [40,45,50], who are breastfeeding infants [34,36] or who have neuropsychiatric conditions such as ADHD, ASD or depression, also appear to benefit more strongly from omega use [29,31,35,39,54].

Most studies focused on the role of omega fatty acids in children and teenagers [30,31,32,33,35,36,37,39]. Of these, the majority were conducted on children with ADHD or ASD [29,31,35,49] whilst two trials focused on mainstream children [38,39]. Omega fatty acids were found to improve ADHD symptoms and fatty acid profiles [31,35,49]. Amongst mainstream school children, 3-months of Omega 3/6 treatment improved reading ability - specifically the clinically relevant “phonologic decoding time” and “visual analysis time”, with children with attention problems again showing particular benefits [39].

In the case of older adults, a number of studies alluded that omega fatty acids could help to protect against neurodegeneration and the chances of developing cognitive impairment [44,48,50,51,52,53]. Similar conclusions have been formulated in other reviews, reflecting the current strength of the evidence base [60,61]. Whilst some research has looked into markers of brain health in the middle years of life, outcomes studied varied and findings were not strong enough to warrant any firm conclusions.

### Limitations and Future Research

When comparing and contrasting study findings, it is important to consider methodological differences. For example, the dosage, length of supplementation and appropriate selection of assessment tools (erythrocyte analysis, where possible) should all be deliberated, especially in varying cultural populations with sufficient statistical power behind studies to allow for investigating interactions between gender and age [43]. Indeed, the present publication only evaluated RCTs which ideally should be at least 16 weeks in duration and measure outcomes of focus immediately during or after supplementation rather than a number of years post-intervention [45]. It is also worth mentioning at this point that movement away from the omega-6 to 3 ratio may now be required in order to fully decipher what an “optimal” omega fatty acid status is [62]. Instead metrics focusing on the shortfalls of EPA and DHA in Western diets should be used as a modern assessor, such as the Omega-3 index [62]. It is also evident that brain-imaging technologies should be used alongside measures such as ‘speed of cognitive performance’ [45].

Future studies should investigate the role of other nutrients such as B vitamins which may interact with omega-3 fatty acids and have a joint role in preventing brain atrophy [63]. Some work conducted with Canadian Inuit’s identified a potential link between low iron intakes (higher serum ferritin levels) and reduced omega-3 fatty acid biosynthesis, indicating that iron deficiency could impact on fatty acid status [64]. Failure to confound or control for factors such as these could be why some trials looking at omega-3 fatty acids and cognition have been unsuccessful [63]. The complex interplay between factors such as apolipoprotein E genotype, gender breakdown, vegetarianism and veganism should also be further studied [45]. Clearly, more work is needed across the middle years of life as we now know that changes in the brain occur before any signs of poor brain health manifest [65]. Dotage, is another life phase worthy of continued exploration given that the number of octogenarians is presently around 15% of the elderly population with numbers projected to exceed the number of children by 2047 [66]. Finally, work looking at the dual effects of omega fatty acid alongside mindfulness strategies could be worthy of exploration given growing interest in this field [67].

## 5. Conclusions

The present article shows that omega-3 supplementation appears to be effective at improving EPA and DHA status. This in turn may have a role to play in maintaining and augmenting brain health, particularly amongst those with low baseline levels or fatty acids or with increased demands such as lactating mothers or those with diagnosed neuropsychiatric conditions. Certainly, given that ageing populations, mental health conditions, and cognitive decline are currently showing no signs of subsiding, it makes good sense to ensure that omega fatty acid levels are optimal across the lifespan be it through dietary or supplement sources.

## Figures and Tables

**Table 1:** Extended roles of omega-3 fatty acids on brain health and beyond.

**Potential Extended Roles:**
Anticoagulation Cardio-protective effects Cognitive function Fetal development Immune function Improved insulin sensitivity in Asians Neuronal function Reduced risk of breast cancer (women) Reduced risk of colorectal cancer (men) Reduced risk of ischemic stroke (men and women) Reduced total stroke risk (women) Retinal function Weight management
**Potential underpinning mechanisms:**
Altered membrane fluidity Anti-inflammatory effects Improved neurogenesis esp. in the hippocampus Modified gene expression Modified intracellular signaling Modulation of ion channels Optimized brain repair mechanisms Protected synaptic transmission Reduced production of pro-inflammatory eicosanoids

Source(s): Endo et al. (2016) [23]; Grant et al. (2016) [24]; Li et al. (2015) [22]; Denis et al. (2013) [25]; Swanson et al. (2012) [26].

**Table 2:** Omega-3 fatty acid supplements and brain health.

Life Stages (Author, Year, Location)	Population (Sample Size, Age, Gender, Health)	Study Design	Intervention (Daily Dose)	Aspect of Brain Health	Key Findings
**Pregnancy/Infancy**					
Brew et al. (2015) Australia [27]	*n* = 616 infants with a family history of asthma.	Ramdomized controlled trial (RCT) from 6 months of infancy to 5 years of age.	500mg tuna fish oil (135 mg DHA, 32 mg EPA, 6% omega-6) per capsule or Sunola oil (control) provided after breastfeeding ceased.	Academic performance, literacy and numeracy assessment.	At 8 years, the proportion of omega-3 fatty acid in plasma was positively associated with the National Assessment Program Literacy and Numeracy score.
Hurtado et al. (2015) Spain [22]	*n* = 110 pregnant women.	RCT from 28th week of pregnancy until 4th month of lactation.	Supplemented group taking 400 mL of a fish oil-enriched dairy drink (-400 mg EPA and DHA) or 400ml control drink.	New-born visual and cognitive development.	Omega-3 LC-PUFA dietary supplement during pregnancy and lactation influenced the mother and newborn’s fatty acid profile and nervonic acid content but did not show effects on visual and cognitive/psychomotor development.
Sherry et al. (2015) USA [28]	*n* = 89 lactating women 4–6 weeks post-partum.	6-week postpartum RCT.	200 mg DHA, 400 mg DHA or placebo for 6 weeks with usual diets.	Maternal and infant plasma fatty acid levels.	Breast milk and maternal plasma DHA were significantly greater with 200 mg and 400 mg DHA compared with placebo which is important for brain development.
Gould et al. (2014) Australia [21]	*n* = 185 term-born children of mothers.	RCT from 20 weeks into pregnancy until measures of attention were assessed after 27 months.	800 mg DHA or a placebo (control).	Child attention and working memory and inhibitory control.	Maternal DHA supplementation during pregnancy does not enhance attention or working memory and inhibitory control in term-born preschoolers.
Mulder et al. (2014) Canada [20]	*n* = 270 pregnant women.	RCT from week 16 of pregnancy to delivery.	400 mg DHA or a placebo.	Central Nervous System development.	Infants in the placebo group were at increased risk of lower language development assessed as words understood and produced at 14 months and words understood and sentences produced at 18 months.
**School age/Teenage**					
Montgomery et al. (2018) UK [29]	*n* = 376 children aged 7–9 years underperforming in reading from primary schools in five counties of the UK.	16-week parallel group, fixed-dose DB RCT.	600 mg DHA (from algal oil), placebo (taste/color matched corn/soybean oil).	Reading, working memory, and behavior.	This RCT did not replicate results of the earlier DOLAB 1 study on the effectiveness of nutritional supplementation with DHA for learning and behavior. Possible reasons are discussed.
Arnold et al. (2017) USA [30]	*n* = 96 7–14 year olds Depression *n* = 72 Bipolar *n* = 23.	12-week placebo-controlled 2X2 design.	1.4 g EPA, 0.2 g DHA 0.27 g other *n*-3.	Mood symptom severity, global function.	2 g *n*-3 increased EPA blood levels sevenfold and DHA levels by half (both *p* < 0.001). Body weight correlated inversely with increased EPA (*r* = −0.52, *p* = 0.004) and DHA (*r* = −0.54, *p* = 0.003) and positively with clinical mood response.
Johnson et al. (2017) Sweden [31]	*n* = 154 mainstream schoolchildren aged 9–10 years.	3-month parallel, randomized, DB, placebo-controlled trial followed by 3-month active treatment for all subjects.	Three Omega 3/6 capsules (Equazen®) twice daily corresponding to a daily dose of 558 mg EPA, 174 mg DHA, and 60 mg gamma-linoleic acid) or identical placebo capsules.	Reading ability, visual analysis, phonological decoding time.	3 months of Omega 3/6 treatment improved reading ability-specifically the clinically relevant ‘phonologic decoding time’ and ‘visual analysis time’-in mainstream schoolchildren. In particular, children with attention problems showed treatment benefits.
Parellada et al. (2017) Spain [32]	*n* = 68 children with ASD > and <12 years.	8-week randomized, crossover, placebo-controlled study.	962 mg for children 1155 mg for adolescents.	Changes in autistic behaviors.	Supplementation with *n*-3 fatty acids might be studied as an add-on to behavioral therapies in ASD. Optimal duration of treatment requires further investigation.
Bos et al. (2015) Netherlands [33]	*n* = 40 boys with ADHD, aged 8–14 years, and 39 matched, typically developing controls.	16-week DB randomized placebo-controlled trial.	Participants consumed 10 g of margarine, enriched with either 650 mg of EPA/DHA each or placebo.	ADHD symptoms, cognitive control	Dietary supplementation with *n*-3 fatty acids reduced symptoms of ADHD, both for individuals with ADHD and typically developing children.
Milte et al. (2015) Australia [34]	*n* = 53 with ADHD.	12-month randomized controlled 3-way crossover trial.	Supplements high in EPA, DHA, or linoleic acid (control) for 4 months each in a crossover design.	Attention, literacy, and behavior.	Increasing erythrocyte DHA and EPA via dietary supplementation may improve behavior, attention, and literacy in children with ADHD.
Portillo-Reyes et al. (2014) Spain [35]	59 children aged 8–12 years.	3-month randomized, DB, treatment and placebo study.	Three capsules providing *n*-3 (each capsule had 60 mg of DHA and 90 mg of EPA). Placebo treatment consisted of soybean oil capsules that looked similar to the active treatment.	Processing speed, attention, memory, language, executive function.	Results show that more than 50% of children in the treatment group had greater improvement in 11 of the 18 neuropsychological variables studied. Processing speed, visual-motor coordination, perceptual integration, attention and executive function showed improvement in more than 70% of the omega-3 supplemented children.
Widen horn-Müller et al. (2014) Germany [36]	95 children, 6–12 years diagnosed with ADHD.	16-week randomized, DB placebo-controlled trial.	720 mg omega-3 fatty acids (600 mg EPA, 120 mg DHA) and 15 mg of vitamin E as antioxidant or placebo treatment.	Behavior, cognitive impairment	Supplementation with the *n*-3 fatty acid mix increased EPA and DHA concentrations in erythrocyte membranes and improved working memory function. Improved working memory correlated significantly with increased EPA, DHA and decreased AA.
Parletta et al. (2013) Australia [37]	*n* = 409 children aged 3–13 years.	20-week RCT.	Omega 3/6 capsules (Equazen®) (providing 750mg DHA plus EPA, and 60mg GLA/school day) for 20 school weeks (Phase 1) followed by one-way crossover to fish oil (Phase 2).	Reading, spelling and non-verbal cognitive development	The treatment group showed improvements in Draw-A-Person compared with the placebo during Phase 1 (*p* = 0.029). The placebo group showed significant within-group improvements after switching to treatment (*p* < 0.001).
**Young adults**					
Giles et al. (2015) USA [38]	*n* = 72 young adults (mean age 20 years).	35 day DB, placebo-controlled design.	2800 mg fish oil or olive oil control.	Mood, cognition, and physiological stress.	Rated anger and confusion increased with stress in the olive oil group, but remained stable in the fish oil group.
Bauer et al. (2014) Australia [39]	*n* = 13 adults 20 to 34 years.	30-day supplementation period. DB counterbalanced, crossover design, with a 30-day washout period between two supplementation periods.	High EPA: DHA formulation (3:1) (400 mg of natural fish oil) with added evening primrose oil (100 mg), whereas the second diet was a high DHA: EPA (4:1) formulation (365.7 mg of natural fish oil). Participants supplemented with 6 capsules daily.	Cognitive performance and functional brain activation.	Following EPA-rich supplementation, participants’ brains worked ‘less hard’ and achieved a better cognitive performance than prior to supplementation.
Stonehouse et al. (2013) New Zealand [40]	*n* = 176 healthy adults 18–45 years, non-smoking and with a low intake of DHA.	6-month randomized, placebo-controlled, DB intervention.	1.16 g DHA or a placebo.	Cognitive performance.	DHA supplementation improved memory and the reaction time of memory in healthy, young adults whose habitual diets were low in DHA. The response was modulated by sex.
**Middle age**					
Külzow et al. (2016) Germany [41]	*n* = 44 cognitively healthy individuals aged 50–75 years.	26-week double-blind placebo-controlled proof-of-concept study.	Either LC-*n*3-FA (2200 mg/day, *n* = 22) or placebo (*n* = 22).	Learning and memory formation	Recall of object locations was significantly better after *n*3-FA supplementation compared with placebo. This study provides further experimental evidence that LC-n3-FA exert positive effects on memory functions in healthy older adults.
Jaremka et al. (2014) USA [42]	*n* = 138 (mean 51 years).	4-month RCT. were randomized.	1.25 g of *n*-3 or 2.5g of *n*-3.	Loneliness-related episodic memory problems.	*n*-3 supplementation attenuates loneliness-related verbal episodic memory declines over time and support the utility of exploring novel interventions for treating episodic memory problems among lonely people.
Witte et al. (2014) Germany [43]	*n* = 65 healthy subjects (50–75 years).	26-week DB randomized interventional study.	Fish oil (2.2 g LC-n3-FA) or placebo.	Cognitive function	Observed a significant increase in executive functions after *n*3-FA compared with placebo (*p* = 0.023). *n*3-FA exerted beneficial effects on white matter microstructural integrity and gray matter volume in frontal, temporal, parietal, and limbic areas primarily of the left hemisphere.
**Older age/Dotage**					
Boespflug et al. (2016) USA [44]	*n* = 140 healthy adults 62–80 years with subjective memory impairment, but not meeting criteria for mild cognitive impairment or dementia.	24-week randomized, DB, placebo-controlled study.	Fish oil (EPA + DHA: 2.4 g/day, *n* = 11) or placebo (corn oil, *n* = 10).	Cortical blood oxygen level-dependent activity during a working memory task.	Dietary fish oil supplementation increases red blood cell *n*-3 content, working memory performance, and blood oxygen level dependent signal in the posterior cingulate cortex during greater working memory load suggesting enhanced neuronal response to working memory challenge.
Bo et al. (2017) China [45]	*n* = 86 adults. Mean age 71 years with mild cognitive impairment.	6-month, randomized, DB, placebo-controlled trial.	*n*-3 PUFAs (480 mg DHA and 720 mg EPA per day, *n* = 44) or placebo (olive oil, *n* = 42) capsules.	Cognitive function.	*n*-3 PUFA supplementation was associated with improved total Basic Cognitive Aptitude Test scores, perceptual speed, space imagery efficiency, and working memory (*p* < 0.01), but not with mental arithmetic efficiency or recognition memory (*p* > 0.05).
Hooper et al. (2017) France [46]	*n* = 183 ≥ 70 years reporting subjective memory complaints, but free from clinical dementia.	Secondary exploratory analysis of the above trial (Andrieu et al., 2017).	*n*-3 FAs (two capsules) providing a total 800 mg DHA and 225 mg EPA or placebo.	Cognitive function domains.	*n*-3 PUFAs may be beneficial for the maintenance of executive functioning in older adults at risk of dementia with low omega-3 index.
Duffy et al. (2015) Australia [47]	*n* = 51 older adults. Mean age 71 years.	12-week RCT.	Four 1000-mg *n*-3 FA supplements (containing EPA 1200 mg plus DHA 800 mg) or placebo.	In vivo glutathione concentration.	Compared with the group given the *n*-3 FA supplements, the placebo group had greater change in the glutathione-to-creatine ratio in the thalamus (*p* = 0.049).
Konagai et al. (2013) Japan [48]	*n* = 45 healthy elderly males, 61–72 years.	12-week randomized, DB, parallel-group comparative study.	2 weeks of treatment with: medium-chain triglycerides as placebo; krill oil, which is rich in *n*-3 PUFAs incorporated in phosphatidylcholine; or sardine oil, which is abundant in *n*-3 PUFAs incorporated in triglycerides.	Cognitive function	*n*-3 PUFAs activated cognitive function in the elderly. This is especially the case with krill oil, in which the majority of *n*-3 PUFAs are incorporated into phosphatidylcholine, causing it to be more effective than sardine oil, in which *n*-3 PUFAs are present as triglycerides.

AA, arachidonic acid; ADHD, attention deficit hyperactivity disorder; ASD, autism spectrum disorder; DB, double-blind; DHA, docosahexaenoic acid; DOLAB, DHA Oxford Learning & Behaviour Study; EPA, eicosapentaenoic acid; FA, fatty acids; GLA, gamma linolenic acid; LC, long chain; PUFA, polyunsaturated fatty acids, RCT, randomized controlled trial.

**Table 3:** Assessment scale used to assess RCT quality.

Publication	Randomization	Method of Randomization Described & Appropriate	Blinding Mentioned	Method of Blinding Described and Appropriate	Withdrawal and Dropout of Subjects Provided	Total Score
**Pregnancy/Infancy**						
Brew et al. (2015) Australia [27]	1	0	1	0	1	3
Hurtado et al. (2015) Spain [22]	1	1	1	1	1	5
Sherry et al. (2015) USA [28]	1	0	1	1	1	4
Gould et al. (2014) Australia [21]	1	1	1	0	1	4
Mulder et al. (2014) Canada [20]	1	1	1	1	1	5
**School age/Teenage**						
Montgomery et al. (2018) UK [29]	1	1	1	1	1	5
Arnold et al. (2017) USA [30]	1	1	1	1	1	5
Johnson et al. (2017) Sweden [31]	1	1	1	1	1	5
Parellada et al. (2017) Spain [32]	1	1	0	0	1	3
Bos et al. (2015) Netherlands [33]	1	1	1	1	1	5
Milte et al. (2015) Australia [34]	1	1	1	0	1	4
Portillo-Reyes et al. (2014) Spain [35]	1	0	0	0	0	1
Widenhorn-Müller et al. (2014) Germany [36]	1	1	1	0	1	4
Parletta et al. (2013) Australia [37]	1	1	1	0	1	4
**Young adults**						
Giles et al. (2015) USA [38]	0	0	1	1	1	2
Bauer et al. (2014) Australia [39]	1	1	1	0	0	3
Stonehouse et al. (2013) [40]	1	1	1	1	1	5
**Middle age**						
Külzow et al. (2016) Germany [41]	1	1	1	1	1	5
Jaremka et al. (2014) USA [42]	1	0	1	1	0	3
Witte et al. (2014) Germany [43]	1	0	0	0	1	2
**Older age/Dotage**						
Boespflug et al. (2016) USA [44]	1	0	1	1	1	4
Bo et al. (2017) China [45]	1	1	1	0	1	4
Hooper et al. (2017) France [46]	1	1	1	1	4	5
Duffy et al. (2015) Australia [47]	1	1	1	1	1	5
Konagai et al. (2013) Japan [48]	1	1	1	1	1	5

Total quality assessment score for which scores range between 1 and 5: with 1 being the lowest quality and 5 being the highest quality. 3 = above average quality.
